# Nodular Scleritis Associated with Herpes Zoster Virus: An Infectious and Immune-Mediated Process

**DOI:** 10.1155/2016/8519394

**Published:** 2016-05-19

**Authors:** Mónica Loureiro, Renata Rothwell, Sofia Fonseca

**Affiliations:** ^1^Vila Nova de Gaia/Espinho Hospitalar Center, R. Conceição Fernandes, 4434-502 Vila Nova de Gaia, Portugal; ^2^Institute of Biomedical Sciences Abel Salazar, University of Porto, Porto, Portugal

## Abstract

*Purpose.* To describe a case of anterior nodular scleritis, preceded by an anterior hypertensive uveitis, which was primarily caused by varicella zoster virus (VZV).* Case Report*. A 54-year-old woman presented with anterior uveitis of the right eye presumably caused by herpetic viral disease and was successfully treated. Two months later, she developed a nodular scleritis and started oral nonsteroidal anti-inflammatory without effect. A complete laboratory workup revealed positivity for HLA-B27; the infectious workup was negative. Therapy was changed to oral prednisolone and an incomplete improvement occurred. Therefore, a diagnostic anterior paracentesis was performed and the polymerase chain reaction (PCR) analysis revealed VZV. She was treated with valacyclovir and the oral prednisolone began to decrease; however, a marked worsening of the scleritis occurred with the reduction of the daily dose; subsequently, methotrexate was introduced allowing the suspension of the prednisolone and led to clinical resolution of the scleritis.* Conclusion.* This report of anterior nodular scleritis caused by VZV argues in favor of an underlying immune-mediated component, requiring immunosuppressive therapy for clinical resolution. The PCR analysis of the aqueous humor was revealed to be a valuable technique and should be considered in cases of scleritis with poor response to treatment.

## 1. Introduction

Scleritis is defined as an inflammation of the sclera. It is classified as anterior or posterior, accordingly to the anatomic site of the disease, and the anterior scleritis can be divided into subtypes: diffuse, nodular, and necrotizing [1, 2].

Immunologically mediated diseases are the main disorders associated with scleritis, some of which are with systemic involvement and other restricted solely to the eye, in an organ-specific autoimmune disease [1, 2]. On the other hand, infections are important but with less common causes, occurring in about 5–10% of all patients presenting with scleral inflammation [3–6]. Attending to the similarity of its presentation, infectious scleritis is often initially managed as autoimmune [7].

Within the infectious agents of scleritis, varicella zoster (VZV) is the virus most often implicated [8]. The direct invasion and the host immune reaction to the virus are the pathophysiological mechanisms proposed. We present an unusual case of infectious scleritis by VZV, which only improved after the immunosuppressor association, supporting the immune-mediated hypothesis.

## 2. Case Report

A 54-year-old Caucasian woman presented with a right painful ocular inflammation and progressive blurring of vision since the few preceding days. At the first visit, best corrected visual acuity (BCVA) of the right eye (RE) was 20/32 and of the left eye (LE) was 20/20. The intraocular pressure (IOP) was 32 mmHg in the RE and 16 mmHg in the left eye. On slit lamp evaluation, her right eye had marked ciliary injection, mild corneal edema with diffuse keratic precipitates, and an anterior chamber reaction (cell grade 2+, flare grade 0); on funduscopic examination there was nothing to report. The left eye was unremarkable. Based on the above clinical features, she was diagnosed with hypertensive anterior uveitis presumably caused by herpetic viral disease. Therefore, treatment with oral valacyclovir (1 g three times daily), topical dexamethasone (1 drop every two hours), topical cyclopentolate, and brinzolamide (twice daily) was prescribed and a progressive improvement of the anterior uveitis was visible.

**Figure 1:**
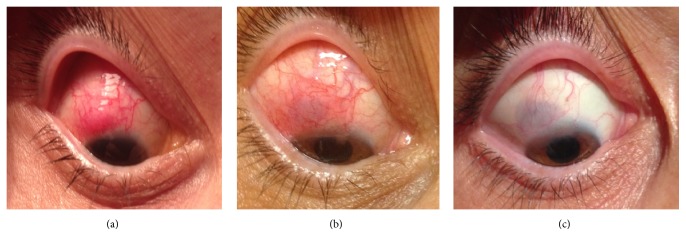
Clinical evolution of anterior nodular scleritis associated with varicella zoster virus. (a) Initial reddish scleral nodule with dilated vessels. (b) Clinical improvement with oral prednisolone 40 mg daily, less hyperemia, and flattening of the nodule. (c) Clinical resolution after methotrexate therapy; the underlying uveal tissue is visible through the thinned sclera giving it a blue-gray appearance.

Two months later and under treatment with oral valacyclovir (500 mg daily), her right eye developed a reddish scleral nodule at 11 o'clock (Figure 1(a)) and tenderness on palpation. At that moment, the biomicroscopy showed control of the prior uveitis, with a transparent cornea and clear anterior chamber, and the fundus remained normal. Given the anterior scleritis she started oral nonsteroidal anti-inflammatory (ibuprofen 600 mg twice daily), without effect. A complete laboratory investigation revealed a normal blood cell count, erythrocyte sedimentation rate, and angiotensin converting enzyme levels. The immunological study was negative for rheumatoid factor, antineutrophil cytoplasmic antibodies (ANCA), and antinuclear antibodies (ANA), although it was positive for HLA-B27. The infectious study, including syphilis, tuberculosis, herpes simplex, and* Borrelia* was negative. Therapy was changed to oral prednisolone 1 mg/Kg/day and an improvement of the scleritis occurred (Figure 1(b)); however, it remained stable for several weeks, without additional improvement. For this reason, a sample of aqueous humor was collected, in the operating room under sterile conditions, and sent for DNA virus analysis (polymerase chain reaction (PCR)) revealing VZV.

After the diagnosis of VZV infection, treatment with valacyclovir was increased (1 g three times daily) and two weeks later the patient began to decrease the oral prednisolone; however, a marked worsening of scleritis occurred with a reduction to a daily dose of 30 mg. Subsequently, weekly methotrexate 15 mg was introduced, allowing the suspension of the prednisolone, with clinical resolution of scleritis in 8 weeks (Figure 1(c)).

## 3. Discussion

Scleritis has a wide spectrum of clinical presentations and etiologic factors, varying from idiopathic to autoimmune or infectious and coursing with variable severity and outcomes [1, 2]. The current case of anterior nodular scleritis presented as a reddish and tender scleral nodule; this clinical picture was indistinguishable from an immune-mediated scleritis and was initially managed as such, emphasizing that diagnosing infectious scleritis often poses difficulties.

The infectious scleritis usually follows accidental or surgical trauma, endophthalmitis, or may occur as an extension of a primary corneal infection [9]. In our case, the patient presented hypertensive anterior uveitis, with diffuse keratic precipitates, preceding the scleritis. This uveitis was probably associated with herpetic viral disease, supported by the response to valacyclovir, and later by the PCR of the aqueous humor which confirmed the presence of VZV. Although VZV infections of the eye are predominantly related to the cornea and ocular adnexa, they have also been implied in cases of scleritis [10].

In a large retrospective study, Gonzalez-Gonzalez et al. found that patients with herpetic scleritis were predominately middle-aged females with unilateral findings and moderate pain [11]; our patient fulfills this profile, although herpetic scleritis is more likely to be diffused than nodular [11]. Usually, infectious scleritis by herpes virus has longer resolution time compared to noninfectious scleritis [12], which is consistent with the clinical course of our patient.

The infectious scleritis due to VZV likely triggered an immune response that also accounted for the inflammation [13], in a patient with a positive HLA-B27. This fact may explain the development of scleritis even under antiviral therapeutic. The virally induced autoimmune reaction is thought to be linked to the release of sequestered self-antigens after virally induced tissue damage and molecular mimicry between viral and self-peptides; this phenomenon involves an immune-complex vasculitis of the eye, similar to other organs such as skin [14].

The clinical resolution of scleritis using immunosuppressive therapy (methotrexate), in a HLA-B27 positive patient, argues in favor of an underlying autoimmune process; on the other hand, the detection of viral DNA in the aqueous humor and the patient's response to antiviral medication strongly support the infectious etiology.

In conclusion, it is critical for eye care professionals to maintain infectious etiologies in their differential diagnosis. This report suggests that cases of nodular scleritis after reactivation of latent VZV infection may also present an immune-mediated component. The PCR analysis of the aqueous humor was revealed to be a valuable technique and should be considered in cases of scleritis with poor response to anti-inflammatory treatment.
